# The use of public transport and contraction of SARS-CoV-2 in a large prospective cohort in Norway

**DOI:** 10.1186/s12879-022-07233-5

**Published:** 2022-03-14

**Authors:** Merete Ellingjord-Dale, Karl Trygve Kalleberg, Mette S. Istre, Anders B. Nygaard, Sonja H. Brunvoll, Linn M. Eggesbø, John Arne Dahl, Eyrun F. Kjetland, Giske Ursin, Arne Søraas

**Affiliations:** 1 Department of Microbiology, Oslo University Hospital, P.O. Box 4950, 0424 Oslo, Norway; 2 Age Labs AS, Oslo, Norway; 3 Centre for Imported and Tropical Diseases, Department of Infectious Diseases, Oslo University Hospital, Oslo, Norway; 4 Discipline of Public Health Medicine, Nelson R Mandela School of Medicine, College of Health Sciences, University of KwaZulu-Natal, Durban, South Africa; 5 Cancer Registry of Norway, Oslo, Norway; 6 Institute of Basic Medical Sciences, University of Oslo, Oslo, Norway; 7 Department of Preventive Medicine, University of Southern California, Los Angeles, CA USA

**Keywords:** Use of public transport, SARS-CoV-2, Prevention, Transmission, Prospective cohort

## Abstract

**Background:**

For many people public transport is the only mode of travel, and it can be challenging to keep the necessary distances in such a restricted space. The exact role of public transportation and risk of SARS-CoV-2 transmission is not known.

**Methods:**

Participants (n = 121,374) were untested adult Norwegian residents recruited through social media who in the spring of 2020 completed a baseline questionnaire on demographics and the use of public transport. Incident cases (n = 1069) had a positive SARS-CoV-2 polymerase chain reaction test registered at the Norwegian Messaging System for Infectious Diseases by January 27, 2021. We investigated the association between the use of public transport and SARS-CoV-2 using logistic regression. Odds ratios (ORs) with 95% confidence intervals (CIs) adjusted for age, calendar time, gender, municipality, smoking, income level, fitness and underlying medical conditions were estimated. Frequency of the use of public transport was reported for 2 week-periods.

**Results:**

Before lockdown, those who tested positive on SARS-CoV-2 were more likely to have used public transport 1–3 times (OR = 1.28, CI 1.09–1.51), 4–10 times (OR = 1.49, CI 1.26–1.77) and ≥ 11 times (OR = 1.50, CI 1.27–1.78, p for trend < 0.0001) than those who had not tested positive.

**Conclusion:**

The use of public transport was positively associated with contracting SARS-CoV-2 both before and after lockdown.

**Supplementary Information:**

The online version contains supplementary material available at 10.1186/s12879-022-07233-5.

## Introduction

In November 2021, the SARS-CoV-2 virus had affected more than 200 countries and there were 226 million confirmed cases worldwide including 4.6 million deaths [1]. Respiratory viruses can be transmitted through surface, droplets and airborne transmission [2]. Public transport is sometimes the only mode of travel for many people and are confined spaces where people mix for extended periods of time. It contains surfaces that are frequently touched that may promote transmission of infectious diseases [3]. There are several studies on microbial infections in train environments [4–7]. One study reported low risk of SARS-CoV-2 transmission by fomites [4] and other studies found an increased risk of airborne transmission during train commute [5–7]. A systematic review of 65 studies on SARS-CoV-2 cluster infections (aggregation of cases) found that public transports (buses, flights, taxis, trains) were one of the major types of SARS-CoV-2 cluster infections [8]. A case–control study of 154 SARS-CoV-2 positive and 160 negative participants, however, found no association between public transport and risk of SARS-CoV-2 [9]. The exact relationship between the use of public transport and risk of SARS-CoV-2 transmission is not known. In a large cohort study in Norway, we investigated the association between the use of public transport and SARS-CoV-2 infection.

## Methods

In this prospective cohort study, participants were recruited between March 28 and April 17, 2020, through social media and nationwide media coverage. Eligible study participants were volunteers not tested for SARS-CoV-2 at the time of recruitment 18 years or older, had a Norwegian identification number and electronic access to the secure national digital governmental identification service. All 122,453 participants signed an electronic consent form and completed an online baseline questionnaire detailing demographics, the use of public transport and other possible risk factors for SARS-CoV-2. The use of public transport was one of many other questions on risk factors. The initial lockdown period in March 2020 lasted 6 weeks and involved closure of kindergartens, schools, gyms, bars, restaurants and major cultural and sports events. After that, there were no additional national school closures, and the population was, largely, only advised on social distancing, with restrictions reinstated from November 5, 2020.

### Exposure

The use of public transport was defined as the use of public or commercial buses, trams, ferries and/or trains. All participants were asked how many times (1–3 times, 4–10 times and 11 times or more) during a 2-week period they used public transport (Additional file 3: Appendix). At baseline (March 28, 2020), there were separate questions regarding the use of public transport before and after March 12, 2020, whether participants had been standing (due to lack of seats) and if they travelled during rush hour. Norway’s initial lockdown started on March 12, 2020.

### Outcome

The outcome was a SARS-CoV-2 positive nasopharyngeal or oropharyngeal swab test determined by real-time polymerase chain reaction. The test was obtained from any accredited Norwegian microbiology laboratory and the test result was reported through the Norwegian Messaging System for Infectious Diseases (MSIS). We included only tests at a time point later than the date of the baseline questionnaire and before January 28, 2021. In Norway, it is mandatory to report all cases of SARS-CoV-2 infections to MSIS. The proportion of new positive SARS-CoV-2 tests by day in Norway at the different time points can be found in Additional file 1: Fig. S2.

### Potential confounders

We defined potential confounders to be age (5 years categories, missing), calendar time (date of questionnaire, continuous), sex (men, women, missing), income (NOK per household and year, below 299,999, 300,000–599,999, 600,000–1,000,000, more than 1,000,000, missing), fitness (very fit, fairly fit, in bad shape, missing), smoking habits (never, former, current, missing), underlying medical conditions (no, yes, missing) and municipality (358 different municipalities, missing).

### Missings

We excluded participants with missing on the use of public transport at baseline. Missing on covariates was included as a separate category in each covariate.

### Bias

In the current study all participants were untested at baseline in order to avoid recall bias and self-selection bias (difference in agreement to participate) between SARS-CoV-2 positive and non-positive participants. Outcome status was obtained from accredited laboratories in order to avoid misclassification of the outcome.

### Statistical analyses

Because of the small losses to follow-up and the low percentage of SARS-CoV-2 infected [10, 11], cumulative incidence was used. The association between the use of public transport before and after the initial lockdown period and subsequent contraction of SARS-CoV-2 was investigated using logistic regression. All individuals who had not contracted SARS-CoV-2 by January 27, 2021, were included as controls. We estimated odds ratios (ORs) with 95% confidence intervals (CIs) adjusting for age, calendar time, gender, municipality, smoking habits, income level, fitness and underlying medical conditions. Trend test was performed by fitting ordinal values corresponding to exposure categories and testing whether the slope coefficient differed from zero. All analyses were performed using Stata (Stata Statistical Software, release 16, Stata Corp., College Station, TX) and R (version 3.6.2). A two-sided p-value of less than 0.05 was considered statistically significant. Sensitivity analyses were performed in health care workers and in non-health care workers, and by sex.

## Results

Of the 122,453 untested volunteers, 1079 were excluded because of missing information on the use of public transport (Additional file 1: Fig. S1). The final study sample consisted of 121,374 untested participants at baseline, of which 1069 were incident SARS-CoV-2 positive cases from March 28, 2020, to January 27, 2021.

**Fig. 1:**
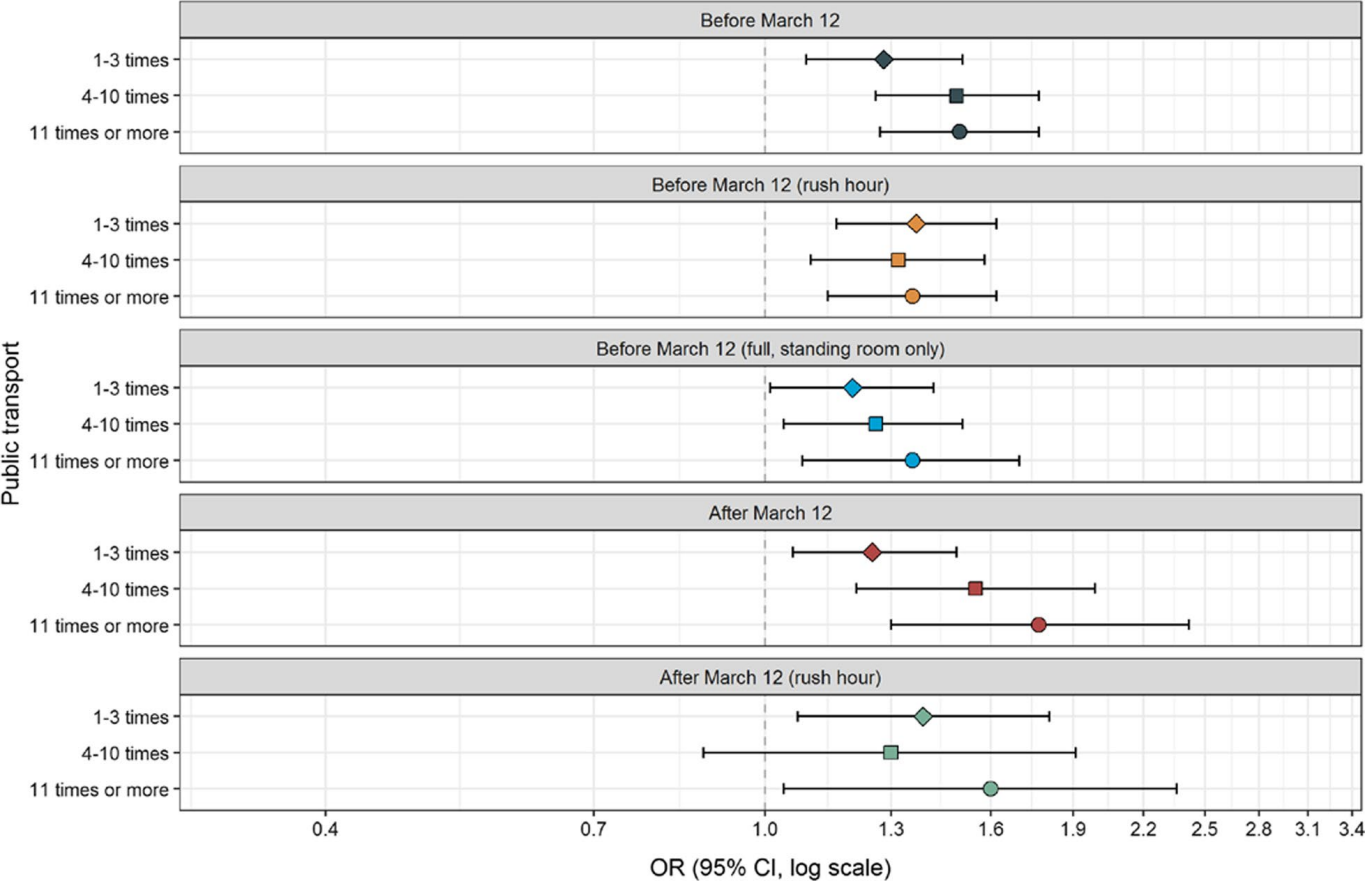
Adjusted odds ratios (OR) and 95% confidence intervals (CI) for the association between the use of public transport and risk of SARS-CoV-2. ^a^Adjusted for age (5-years categories, missing), calendar time (continuous) gender (men/women, missing) smoking habits (never, ever, missing), municipality (358 different, missing) income level per household (< 299,999, 300,000–599,999, 600,000–1,000,000, > 1,000,000 NOK, missing) fitness (very fit, fairly fit, in bad shape, missing), underlying medical condition (no, yes, missing). Likelihood ratio test comparing a model with the use of public transport before lockdown with a model with the use of public transport after lockdown p = 0.004

**Fig. 2:**
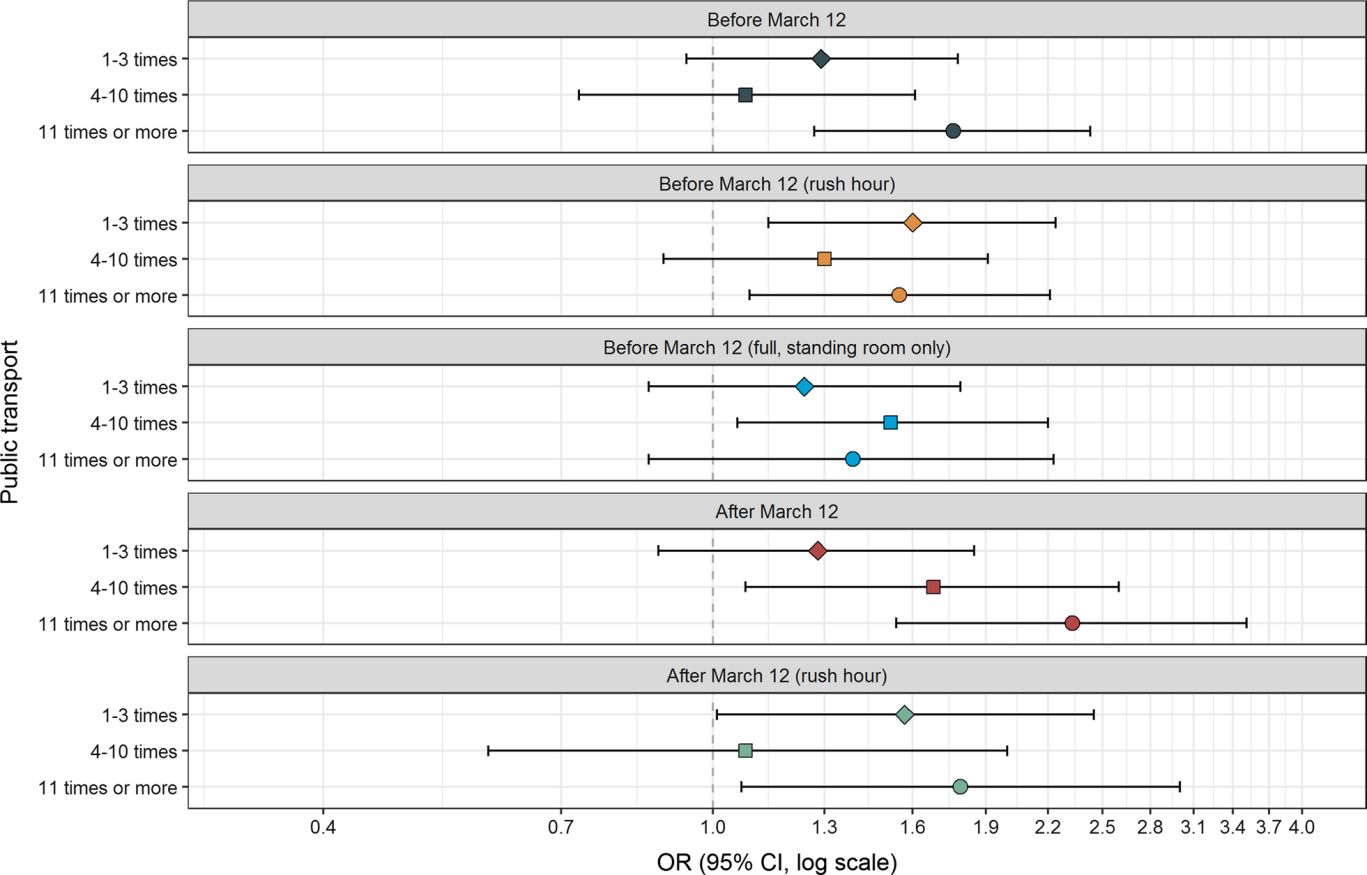
Adjusted odds ratios (OR) and 95% confidence intervals (CI) for the association between the use of public transport and risk of SARS-CoV-2 among health care workers (n = 22,037). ^a^Adjusted for age (5-years categories, missing), calendar time (continuous) gender (men/women, missing) smoking habits (never, ever, missing), municipality (358 different, missing) income level per household (< 299,999, 300,000–599,999, 600,000–1,000,000, > 1,000,000 NOK, missing) fitness (very fit, fairly fit, in bad shape, missing), underlying medical condition (no, yes, missing). Likelihood ratio test comparing a model with the use of public transport before lockdown with a model with the use of public transport before and after lockdown p = 0.005

**Fig. 3:**
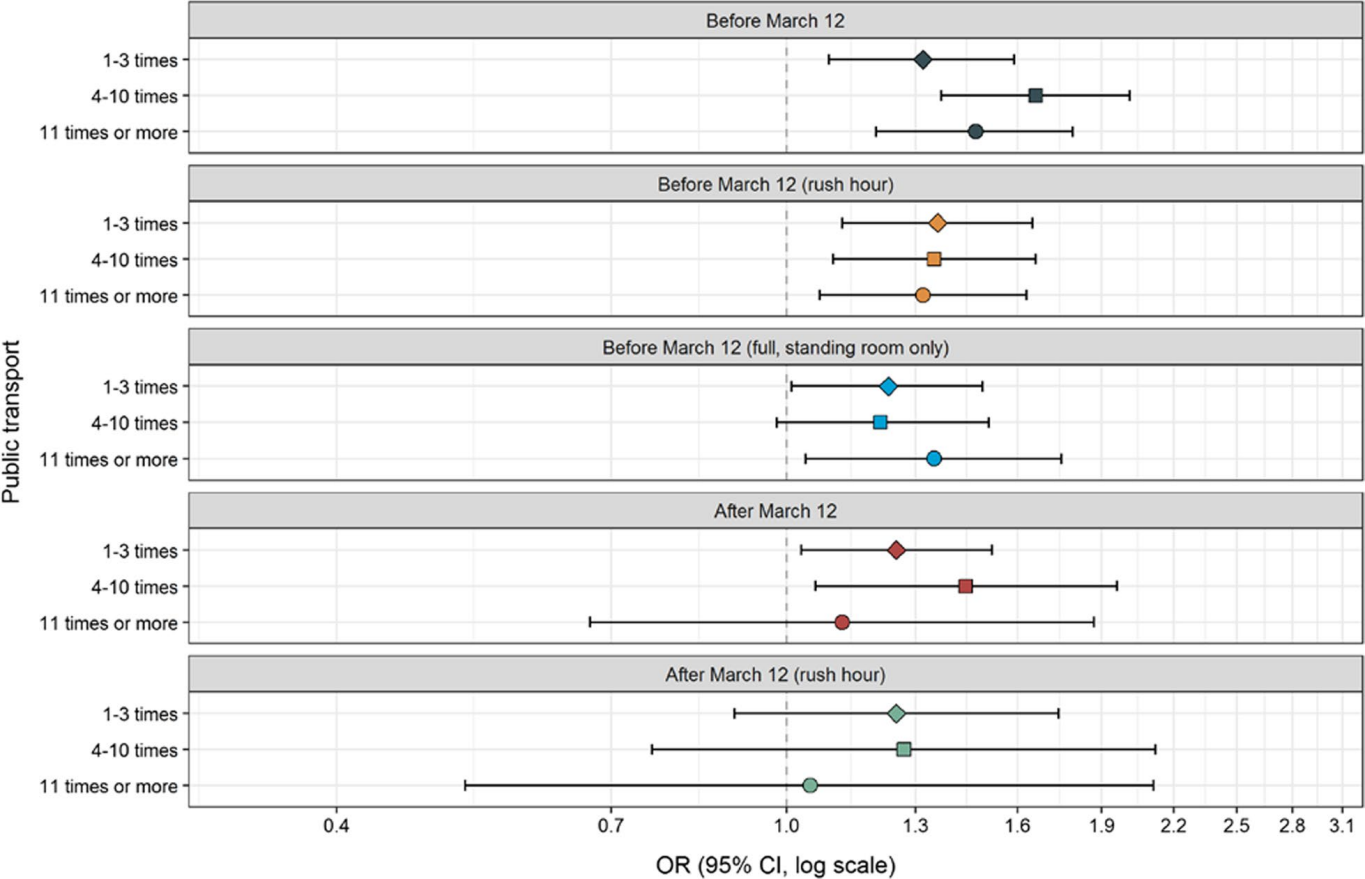
Adjusted odds ratios (OR) and 95% confidence intervals (CI) for the association between the use of public transport and risk of SARS-CoV-2 among non-health care workers (n = 97,962). ^a^Adjusted for age (5-years categories, missing), calendar time (continuous) gender (men/women, missing) smoking habits (never, ever, missing), municipality (358 different, missing) income level per household (< 299,999, 300,000–599,999, 600,000–1,000,000, > 1,000,000 NOK, missing) fitness (very fit, fairly fit, in bad shape, missing), underlying medical condition (no, yes, missing)

Table 1 shows that SARS-CoV-2 positive cases were younger [Mean age (Standard deviation (SD) = 43 (13.7)] than controls [Mean age (SD) = 46 (13.7), Mann Whitney test p < 0.0001], and participants reporting no underlying medical conditions were more likely to acquire SARS-CoV-2 than controls. There were no differences between SARS-CoV-2 positive cases and controls regarding the possible confounders sex, income, fitness or smoking habits. There was a clear association between the use of public transport before lockdown and subsequent testing positive to SARS-CoV-2. The ORs of SARS-CoV-2 were elevated in those who used public transport 1–3 times (OR = 1.28, CI 1.09–1.51), 4–10 times (OR = 1.49, CI 1.26–1.77) and ≥ 11 times in 2 weeks (OR = 1.50, CI 1.27–1.78, p for trend < 0.0001, Fig. 1, Additional file 2: Table S1). After lockdown, the use of public transport was still associated with elevated ORs of SARS-CoV-2 both for the use of public transport 4–10 times (OR = 1.55, CI 1.21–1.99) and ≥ 11 times in 2 weeks (OR = 1.77, CI 1.30–2.42, likelihood-ratio test comparing before and after lockdown p = 0.004). Calculations of the population attributable fraction showed that before lockdown 19% of the SARS-CoV-2 positive cases could have been avoided if no one used public transport (Additional file 2: Table S1). Whereas after lockdown, 7% of the SARS-CoV-2 positive cases could have been avoided. There was no significant difference for the use of public transport during rush hour before and after lockdown (likelihood-ratio p = 0.08, Fig. 1). When we stratified the analyses by sex, there was no difference in the association between the use of public transport and SARS-CoV-2 (results not shown). When stratifying analyses into health care workers and non-health care workers, the results remained the same (Figs. 2 and 3). We observed a higher OR of SARS-CoV-2 for the use of public transport after lockdown compared to before lockdown in health care workers (likelihood-ratio p = 0.005).

**Table 1:** Descriptive statistics (n /%) on covariates in SARS CoV-2 positive cases (n = 1069) and non-positive SARS CoV-2 participants (n = 120,305)^a^

Variable	SARS-CoV-2 positive cases		Non-positive SARS-CoV-2 participants	
	Mean	SD	Mean	SD
Calendar time (date)	31.03.2020	9.5	01.04.2020	12.5
Age (years)	43	13.7	46	13.7
	n	%	n	%
P-value^b^	< 0.0001			
18–25	129	12	6151	5
26–30	117	11	10,237	8
31–35	116	11	13,313	11
36–40	114	11	13,989	12
41–45	123	12	14,728	12
46–50	130	12	15,595	13
51–55	126	12	14,168	12
56–60	101	9	10,712	9
61–65	54	5	9076	8
66–70	26	2	6722	6
> 70	30	3	5300	4
Missing	3	0	314	0
	n	%	n	%
Sex				
Men	318	30	36,460	30
Women	749	70	83,552	70
Missing	2	0.2	293	0.2
P-value^c^	0.86			
Income (NOK per household and year)				
Below 299,999	47	4	4058	3
300,000–599,999	165	15	19,969	17
600,000–1,000,000	272	26	31,305	26
More than 1,000,000	401	38	44,251	37
Missing	184	17	20,722	17
P-value^c^	0.35			
Fitness				
Very fit	390	36	39,709	33
Fairly fit	599	56	69,641	58
In bad shape	80	8	10,864	9
Missing	0	0	91	0
P-value^c^	0.04			
Smoking habits				
Never	577	54	63,044	52
Former	406	38	45,549	38
Current	61	6	9111	8
Missing	25	2	2602	2
P-value ^c^	0.14			
Underlying medical conditions^**d**^				
No	626	59	63,425	53
Yes	283	26	36,792	30
Missing	160	15	20,088	17
P-value^c^	0.001			

^a^The statistical analyses were also adjusted for municipality (358 different municipalities)

^b^Mann-Whitney test comparing equality of medians

^c^Chi-squared test

^d^Chronic heart disease, high blood pressure, chronic lung disease (not asthma), asthma, diabetes, receiving immunodeficiency treatment, cancer (under treatment)

## Discussion

Taking a bus or a train was associated with SARS-CoV-2 infection both before and after lockdown. There was some evidence of a dose–response relationship between the use of public transport and SARS-CoV-2 infection, even after controlling for other risk factors. Furthermore, morning and afternoon congestion on public transport seem to be a risky endeavor.

This investigation is a large prospective cohort study on SARS-CoV-2. The majority of participants were women, younger than 50 years old, and had a higher income. This makes the results less generalizable to men, older than 50 years with a lower income. However, when we stratified the analyses by sex, there were no large differences in the association between the use of public transport and SARS-CoV-2 infection. Although we adjusted for income, we cannot preclude the possibility of residual confounding or unmeasured confounding by socioeconomic status.

Many of the participants were health professionals who had very easy access to tests from the beginning of the pandemic. We found that the use of public transport was positively associated with contraction of SARS-CoV-2, both in health care workers and in non-health care workers. We do not know the reason why we observed slightly higher ORs among health care workers than non-health care workers both before and after lockdown. It is possible that health care workers were more likely to use public transport than non-health care workers.

The baseline questionnaire was administered prior to SARS-CoV-2 diagnosis. The SARS-CoV-2 positive status was obtained from MSIS. This makes the study less prone to misclassification of the outcome. However, we cannot exclude the possibility that some individuals could have been asymptomatic, therefore never tested and misclassified, but this misclassification would probably be non-differential because the misclassification of the SARS-CoV-2 status is not related to the use of public transport.

Consistent with our finding of an association between the use of public transport and transmission of SARS-CoV-2, a systematic review on SARS-CoV-2 cluster infections concluded that public transport was one of the important cluster infection [8]. Similarly, studies of train passengers in China indicated that there was a transmission risk of SARS-CoV-2 among passengers, and that the relative risk depended on the seat location (social distance) and travel duration [12, 13]. SARS-CoV-2 transmission was identified during a bus journey early in the pandemic in China [14]. A study of case reports in 320 municipalities in China identified 318 outbreaks with three or more cases. All the outbreaks were associated with being in an indoor environment. Home-based outbreaks were the dominant category, followed by transport-based outbreaks [15].

One limitation of the current study is that we did not take any air or surface samples of the participants. However, it is important to elucidate the routes of transmission. A review on 14 experimental studies reported a strong likelihood of airborne transmission of SARS-CoV-2 in indoor air [16]. Another cross-sectional study from Iran on 28 SARS-CoV-2 air samples on subways, buses and trains concluded that vehicles were contaminated with SARS-CoV-2 [17]. Further, a case study on 244 individuals in China, reported of airborne transmission on a bus trip [18].

In contrast to the current study, a study in outpatient health care facilities in Nashville, the United States, found no association between the use of public transport and risk of SARS-CoV-2 [9]. The latter study was much smaller than the current study and the controls were symptomatic, indicating exposure to others (with any infection) in both groups [19]. In Norway, people generally did not use face masks during the first part of the pandemic and were therefore potentially more exposed to SARS-CoV-2 transmission through air. However, from August 14, 2020, public health authorities recommended face masks during public transport if social distancing (1 m) was impossible, and from November 2020 it was commonly used.

In the current study we found a relatively higher OR of SARS-CoV-2 infection after lockdown compared to before lockdown. This could indicate that there was a higher transmission rate in the general population after lockdown. Explanations of this difference could be that “the pre- lockdown” period was shorter (2 weeks before March 12) than “the post-lockdown” period (the two previous weeks), and that testing was more extensive after lockdown than before lockdown. We also observed that fewer participants used public transport after lockdown compared to before lockdown. This is in line with the finding in a study from Australia [20] and from the UK ^21^ asking about travel activity at different time points during the pandemic.

## Conclusion

The current study found that the use of public transport is positively associated with contraction of SARS-CoV-2. We cautiously suggest that social distancing, use of hand sanitizer and face masks could lessen the risk of SARS-CoV-2 on public transport.

## Supplementary Information

**Additional file 1.** Supplementary Figure S1. Flow chart of the Norwegian Covid-19 cohort study.

**Additional file 2.** Supplementary Table S1. Adjusted odds ratio and 95% confidence interval (CI) for the association between the use of public transport (baseline only) and risk of SARS-CoV-2.

**Additional file 3.** Supplementary Figure S2. Proportion of new positive SARS-CoV-2 tests in Norway during different time points indicating different test criteria.

## Data Availability

The datasets used and/or analyzed during the current study available from the corresponding author on reasonable request.

## References

[1] World Health Organization. WHO Coronavirus (COVID-19) Dashboard, https://covid19.who.int/. 2021.

[2] The Lancet Respiratory, M (2020). COVID-19 transmission-up in the air. Lancet Respir Med.

[3] Shen J (2020). Prevention and control of COVID-19 in public transportation: experience from China. Environ Pollut.

[4] Mondelli MU, Colaneri M, Seminari EM, Baldanti F, Bruno R (2021). Low risk of SARS-CoV-2 transmission by fomites in real-life conditions. Lancet Infect Dis.

[5] Coleman KK (2018). Bioaerosol sampling for respiratory viruses in Singapore’s mass rapid transit network. Sci Rep.

[6] Furuya H (2007). Risk of transmission of airborne infection during train commute based on mathematical model. Environ Health Prev Med.

[7] Gosce L, Johansson A (2018). Analysing the link between public transport use and airborne transmission: mobility and contagion in the London underground. Environ Health.

[8] Liu T (2020). Cluster infections play important roles in the rapid evolution of COVID-19 transmission: a systematic review. Int J Infect Dis.

[9] Fisher KA (2020). Community and close contact exposures associated with COVID-19 among symptomatic adults >/=18 years in 11 outpatient health care facilities—United States, July 2020. MMWR Morb Mortal Wkly Rep.

[10] Aschengrau A, Seage III, GR. Essentials of epidemiology in public health. 2nd edn, 2008.

[11] Szklo M, Nieto FJ. Epidemiology: beyond the Basics. 4th edn, (Jones & Bartlett Learning, 2018).

[12] Zhang Y, Zhang A, Wang J (2020). Exploring the roles of high-speed train, air and coach services in the spread of COVID-19 in China. Transp Policy (Oxf).

[13] Hu M (2021). Risk of Coronavirus Disease 2019 transmission in train passengers: an epidemiological and modeling study. Clin Infect Dis.

[14] Shen Y (2020). Community outbreak investigation of SARS-CoV-2 transmission among bus riders in Eastern China. JAMA Intern Med.

[15] Qian H (2021). Indoor transmission of SARS-CoV-2. Indoor Air.

[16] Noorimotlagh Z, Jaafarzadeh N, Martinez SS, Mirzaee SA (2021). A systematic review of possible airborne transmission of the COVID-19 virus (SARS-CoV-2) in the indoor air environment. Environ Res.

[17] Hadei M (2021). Presence of SARS-CoV-2 in the air of public places and transportation. Atmos Pollut Res.

[18] Luo K (2020). Transmission of SARS-CoV-2 in public transportation vehicles: a case study in Hunan Province, China. Open Forum Infect Dis.

[19] Zhen J (2020). Transmission of respiratory viruses when using public ground transport: a rapid review to inform public health recommendations during the COVID-19 pandemic. S Afr Med J.

[20] Beck MJ, Hensher DA, Wei E (2020). Slowly coming out of COVID-19 restrictions in Australia: implications for working from home and commuting trips by car and public transport. J Transp Geogr.

[21] Vickerman R (2021). Will Covid-19 put the public back in public transport? A UK perspective. Transp Policy (Oxf).

