# Supplementary material for: The use of public transport and contraction of SARS-CoV-2 in a large prospective cohort in Norway
Source: BMC Infect Dis. 2022 Mar 14;22:252. doi: 10.1186/s12879-022-07233-5 (PMC8919146; doi:10.1186/s12879-022-07233-5)
Supplement: Supplementary file 1 — Additional file 1. Supplementary Figure S1. Flow chart of the Norwegian Covid-19 cohort study. [file 12879_2022_7233_MOESM1_ESM.pdf]

Supplementary Figure 1. Flow chart Norwegian Covid-19 cohort study

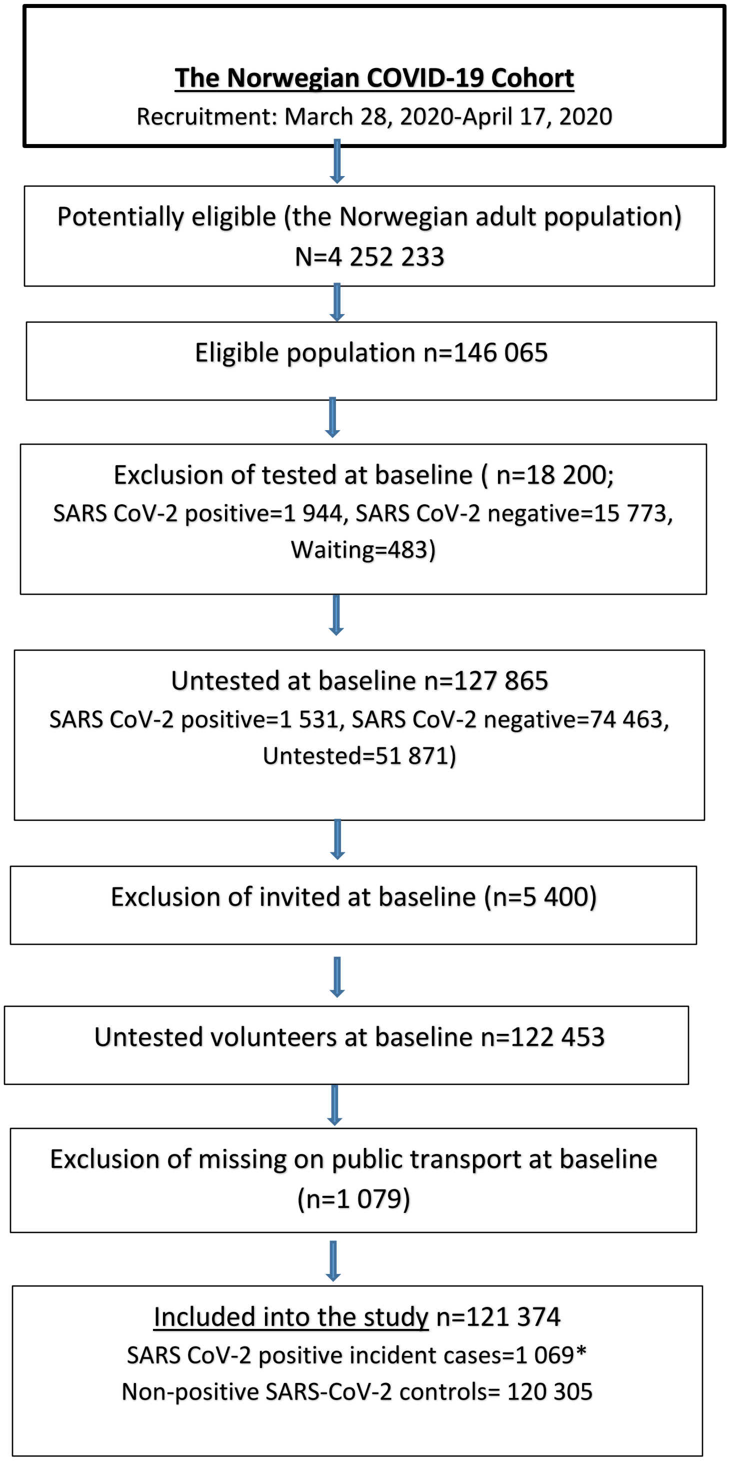

* SARS CoV-2 positivity status obtained from The Norwegian Mandatory System for Reporting of Infectious Diseases
