# Supplementary material for: The use of public transport and contraction of SARS-CoV-2 in a large prospective cohort in Norway
Source: BMC Infect Dis. 2022 Mar 14;22:252. doi: 10.1186/s12879-022-07233-5 (PMC8919146; doi:10.1186/s12879-022-07233-5)
Supplement: Supplementary file 2 — Additional file 2. Supplementary Table S1. Adjusted odds ratio and 95% confidence interval (CI) for the association between the use of public transport (baseline only) and risk of SARS-CoV-2. [file 12879_2022_7233_MOESM2_ESM.docx]

**Supplementary materials**

**Supplementary Table S1.** Adjusted odds ratio and 95% confidence interval (CI) for the association between the use of public transport (baseline only) and risk of SARS-CoV-2^a^.

|  | **SARS-CoV-2 positive**  **cases (n (%))** | **Controls**  **(n (%))** | **Adj. Odds ratio** | **95% CI** | **P for trend** |  |
| --- | --- | --- | --- | --- | --- | --- |
| **BEFORE LOCKDOWN (last 2 weeks)** | | | | | |  |
| **Any public transport** | | | | | |  |
| None | 385 (36%) | 54997 (46%) | Ref |  |  |  |
| 1-3 times | 247 (23%) | 27141 (23%) | 1.28 | 1.09-1.51 |  |  |
| 4-10 times | 207 (19%) | 18921 (15%) | 1.49 | 1.26-1.77 |  |  |
| 11 times or more | 230 (22%) | 19246 (16%) | 1.50 | 1.27-1.77 | <0.0001 |  |
| PAF (min, max) | 0.19 (0.13-0.24) |  |  |  |  |  |
| **Public transport during rush hour** | | | | | |  |
| None | 570 (53%) | 75317 (63%) | Ref |  |  |  |
| 1-3 times | 183 (17%) | 17049 (14%) | 1.37 | 1.16-1.62 |  |  |
| 4-10 times | 157 (15%) | 14264 (12%) | 1.32 | 1.10-1.58 |  |  |
| 11 times or more | 159 (15%) | 13675 (11%) | 1.36 | 1.14-1.62 | <0.0001 |  |
| **Public transport when standing room only** |  |  |  |  |  |  |
| None | 673 (63%) | 84473 (70%) | Ref |  |  |  |
| 1-3 times | 166 (16%) | 16220 (14%) | 1.20 | 1.01-1.42 |  |  |
| 4-10 times | 141 (13%) | 12502 (10%) | 1.26 | 1.04-1.51 |  |  |
| 11 times or more | 89 (8%) | 7110 (6%) | 1.36 | 1.08-1.70 | <0.0001 |  |
| **DURING LOCKDOWN (last 2 weeks)** | | | | | |  |
| **Any public transport** | | | | | |  |
| None | 796 (74%) | 98081 (82%) | Ref |  |  |  |
| 1-3 times | 160 (15%) | 14582 (12%) | 1.25 | 1.06-1.49 |  |  |
| 4-10 times | 70 (7%) | 5022 (4%) | 1.55 | 1.21-1.99 |  |  |
| 11 times or more | 43 (4%) | 2620 (2%) | 1.77 | 1.30-2.42 | <0.0001 |  |
| PAF (min, max) 0.07 (0.05-0.10)  **Public transport during rush hour** | | | | | |  |
| None | 957 (89%) | 111794 (93%) | Ref |  |  |  |
| 1-3 times | 61 (6%) | 4732 (4%) | 1.39 | 1.07-1.81 |  |  |
| 4-10 times | 27 (3%) | 2183 (2%) | 1.30 | 0.88-1.91 |  |  |
| 11 times or more | 24 (2%) | 1596 (1%) | 1.60 | 1.04-2.36 | 0.002 |  |
| **Public transport when standing room only** ^b^ |  |  |  |  |  |  |
| None | 1025 (96%) | 116 911 (97%) | Ref |  |  |  |
| 1-3 times | 31 (3%) | 2284 (2%) | 1.41 | 0.98-2.02 |  |  |
| 4 times or more | 13 (1%) | 1110 (1%) | 1.20 | 0.69-2.08 | 0.12 |  |
| ^a^ Adjusted for age (5-years categories, missing), calendar time (continuous), gender (men/women, missing) smoking habits (never, ever, missing), municipality (358 different, missing) income level per household (< 299 999, 300 000-599 999, 600 000-1000 000, >1000 000 NOK, missing) fitness (very fit, fairly fit, in bad shape, missing), underlying medical condition (no, yes, missing).  ^b^ Due to low numbers in the highest category, ≥11 times was combined with ≥4 times. | | | | | |  |
|  | | | | | |  |

**Supplementary Figure S2**.

Proportion of new positive SARS-CoV-2 tests in Norway during different time points indicating different test criteria ^a^.


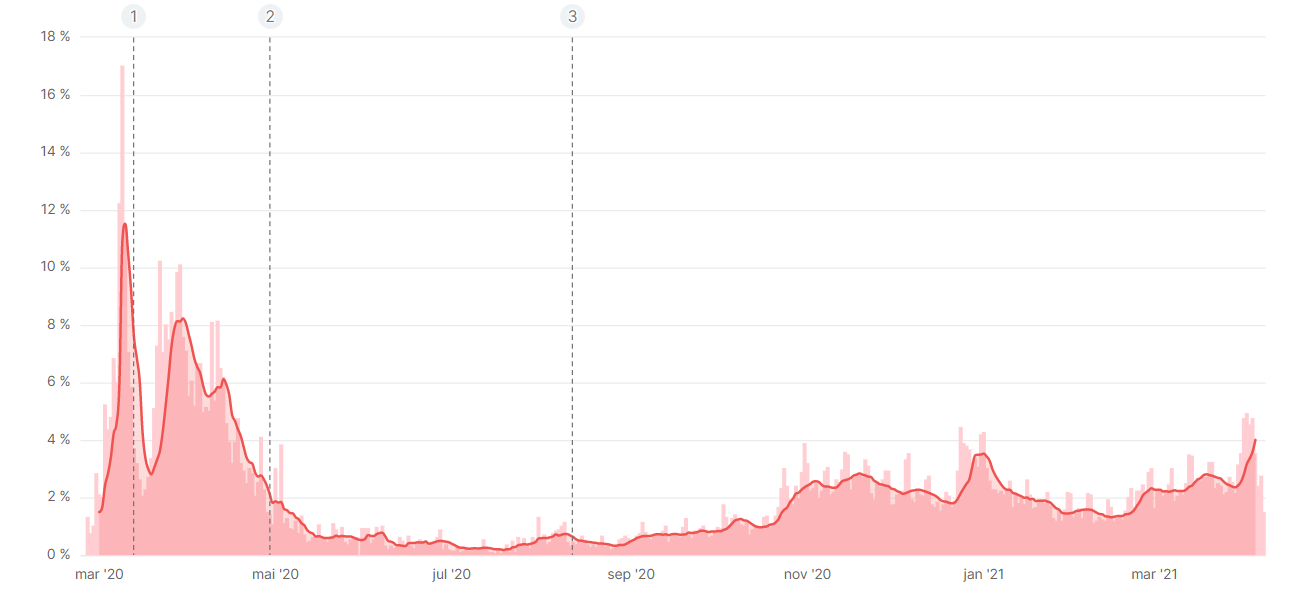


^a^ Source: The Norwegian Institute of Public Health

Test criteria

1. March 13, 2020: Testing was reserved for certain patient groups and health care workers with respiratory symptoms.

2. April 29, 2020: Testing was extended to include everyone that a doctor suspected to have been infected by SARS-CoV-2.

3. August 12, 2020: Testing was even further extended and medical evaluation is no longer a requirement to be tested.
