# Supplementary material for: The use of public transport and contraction of SARS-CoV-2 in a large prospective cohort in Norway
Source: BMC Infect Dis. 2022 Mar 14;22:252. doi: 10.1186/s12879-022-07233-5 (PMC8919146; doi:10.1186/s12879-022-07233-5)
Supplement: Supplementary file 3 — Additional file 3. Supplementary Figure S2. Proportion of new positive SARS-CoV-2 tests in Norway during different time points indicating different test criteria. [file 12879_2022_7233_MOESM3_ESM.docx]

The Risk of Contracting Coronavirus in Norway – At Work and in the Community

Page 1

The information that you provide in this form will be stored separately from your identity.

Anonymized ID

Field completed automatically

How did you hear about this study?

Multiple answers are possible.

I was invited via SMS, email, letter or in person

Through the media

Other

Elaborate on how you found out about this study:

This question only appears if the option 'Other' is selected in the question

'How did you hear about this study?

How anxious are you about being infected by coronavirus (COVID-19)?

Very

Somewhat

Not at all

I have been tested for coronavirus. *

Yes, at least one test came back positive and I have/had coronavirus Yes, but the result was negative

Yes, and I am waiting for the result

No, I have not been tested

Date of first test?

This question only appears if the option 'Yes, at least one test came back positive and I have/had coronavirus', 'Yes, but the result was negative' or 'Yes, and I am waiting for

the result' is selected in the question 'I have been tested for coronavirus'.

Date of second test if applicable?

This question only appears if the option 'Yes, at least one test came back positive and I have/had coronavirus', 'Yes, but the result was negative' or 'Yes, and I am waiting for

the result' is selected in the question 'I have been tested for coronavirus'.

Leave blank if not applicable.

Dates and results of any subsequent tests:

This question only appears if the option 'Yes, at least one test came back positive and I have/had coronavirus', 'Yes, but the result was negative' or 'Yes, and I am waiting for

the result' is selected in the question 'I have been tested for coronavirus'.

Leave blank if not applicable.

I have symptoms of a coronavirus infection.

Yes

No

Don’t know

Check off every symptom that you have had over the course of the last three weeks:

Multiple answers are possible.

Fever

High fever (over 39)

Shortness of breath

Cough

Tiredness/exhaustion

Muscle pains Sore throat

Impaired sense of smell/taste

Blocked or runny nose

Headache

Stomach pains/nausea/diarrhea

Other symptoms

No symptoms

Elaborate on what other symptoms you have:

This question only appears if the option 'Other symptoms' is selected in the question 'Check off every symptom that you have had over the course of the last three weeks:'

Number of days you have had any of these symptoms:

This question only appears if at least one of the options 'Blocked or runny nose', 'Tiredness/exhaustion', 'Stomach pains/nausea/diarrhea', 'Shortness of breath', 'High

fever (over 39)', 'Muscle pains', 'Sore throat', 'Impaired sense of smell/taste',

'Other symptoms', 'Headache', 'Fever' or 'Cough' are selected in the question

'Check off every symptom that you have had over the course of the last three weeks:'

Total 0–21 days.

When did you first experience these symptoms?

This question only appears if at least one of the options 'Blocked or runny nose', 'Tiredness/exhaustion', 'Stomach pains/nausea/diarrhea', 'Shortness of breath', 'High

fever (over 39)', 'Muscle pains', 'Sore throat', 'Impaired sense of smell/taste',

'Other symptoms', 'Headache', 'Fever' or 'Cough' are selected in the question

'Check off every symptom that you have had over the course of the last three weeks:'

Have you had close contact with anybody with confirmed coronavirus over the last three weeks?

Yes

No

Don’t know

Have you had close contact with anybody with probable coronavirus over the last three weeks?

Yes

No

Don’t know

Do you know who you were likely infected by?

This question only appears if the option 'Yes, at least once test came back positive and I have/had coronavirus' is selected in the question 'I have been tested for

coronavirus.'

Yes

No

Do you know how you were infected with coronavirus?

This question only appears if the option 'Yes, at least once test came back positive and I have/had coronavirus' is selected in the question 'I have been tested for

coronavirus.'

Yes

No

Describe how you likely got the infection:

This question only appears if the option 'Yes, at least once test came back positive and I have/had coronavirus' is selected in the question 'I have been tested for

coronavirus.'

Date of presumed infection:

This question only appears if the option 'Yes, at least once test came back positive and I have/had coronavirus' is selected in the question 'I have been tested for

coronavirus.'

Where did you likely get infected?

This question only appears if the option 'Yes, at least once test came back positive and I have/had coronavirus' is selected in the question 'I have been tested for

coronavirus.'

Only one answer is possible.

Norway

Italy

Austria

Another country

Write in the name of the country:

This question only appears if the option 'Another country' is selected in the question

'Where did you likely get infected?'

Are you currently hospitalized?

Yes, due to coronavirus

Yes, but due to an illness/condition other than coronavirus No

What illness/condition are you in hospital with?

This question only appears if the option 'Yes, but due to an illness/condition other than coronavirus' is selected in the question 'Are you currently

hospitalized?'

When were you admitted to hospital?

This question only appears if the option 'Yes, due to coronavirus' or 'Yes, but due to an illness/condition other than coronavirus' is selected in

the question 'Are you currently hospitalized?'

I have previously been hospitalized due to coronavirus.

Yes

No

Don’t know

I am currently in quarantine or home isolation due to coronavirus.

Everyone in Norway has been recommended to self-isolate socially. Quarantine means that you have been ordered to quarantine or that you are part of a group in quarantine in accordance with the national guidelines.

Yes

No

Don’t know

I am in quarantine because:

This question only appears if the option 'Yes' is selected in the question 'I am currently in quarantine or home isolation due to coronavirus'.

Choose the option that best matches your circumstances. Everyone in Norway has been recommended to isolate themselves socially. Quarantine means that you have been ordered to quarantine or that you are part of a group in quarantine in accordance with the national guidelines.

I have been in close contact with an infected person I have traveled to a risk area

I have traveled abroad, though not to a known risk area I was exposed in a work context

I am a healthcare worker and have been put in quarantine following a risk situation Other

Elaborate on the reason you are in quarantine:

This question only appears if the option 'Other' is selected in the question 'I am in quarantine because:'

Date you entered quarantine or began home isolation:

This question only appears if the option 'Yes' is selected in the question 'I am currently in quarantine or home isolation due to coronavirus'.

Page break

Do you work in the healthcare sector?

Page 2

Yes

No

Don’t know

What sort of healthcare job do you have?

This question only appears if the option 'Yes' is selected in the question 'Do you work in the healthcare sector?'

Nurse

Auxiliary

Nurse

Physician

Medical Secretary

Office staff

Cleaner

Porter

Radiographer

Medical laboratory assistant

Physiotherapist

Other

Tell us what job you do:

This question only appears if the option 'Other' is selected in the question

'What sort of healthcare job do you have?'

Do you have direct contact with patients?

This question only appears if the option 'Yes' is selected in the question 'Do you work in the healthcare sector?'

Daily Weekly

Less than weekly Never

Where do you work?

This question only appears if the option 'Yes' is selected in the question 'Do you work in the healthcare sector?'

In an Emergency Department

In an Infectious Disease Department

On an intermediate/intensive-care ward

In a newly established COVID Department

Municipal Health Services Other

This question only appears if the option 'Yes' is selected in the question 'Do you work in the healthcare sector?'

Questions about infection control at your work

I know how to use personal protective equipment in my job.

This question only appears if the option 'Yes' is selected in the question 'Do you work in the healthcare sector?'

Rate the extent to which you agree on a scale of 0 (completely disagree) to 7 (completely agree).

0 completely disagree

1

2

3

4

5

6

7 completely agree

Don’t know

There is enough personal protective equipment available where I work.

This question only appears if the option 'Yes' is selected in the question 'Do you work in the healthcare sector?'

Rate the extent to which you agree on a scale of 0 (completely disagree) to 7 (completely agree).

0 completely disagree

1

2

3

4

5

6

7 completely agree Don’t know

I have been well trained in how to use personal protective equipment.

This question only appears if the option 'Yes' is selected in the question 'Do you work in the healthcare sector?'

Rate the extent to which you agree on a scale of 0 (completely disagree) to 7 (completely agree).

0 completely disagree

1

2

3

4

5

6

7 completely agree

Don’t know

Personal protective equipment must be rationed where I work.

This question only appears if the option 'Yes' is selected in the question 'Do you work in the healthcare sector?'

Rate the extent to which you agree on a scale of 0 (completely disagree) to 7 (completely agree).

0 completely disagree

1

2

3

4

5

6

7 completely agree

Don’t know

The following personal protective equipment must be used with coronavirus:

This question only appears if the option 'Yes' is selected in the question 'Do you work in the healthcare sector?'

Multiple answers are possible.

Contact protective equipment

Droplet protective equipment

Droplet protective equipment witheye protection.

Respiratory protective equipment (P3 respirator)

Don’t know

Have you had to reuse personal protective equipment that was originally intended for single use only?

This question only appears if the option 'Yes' is selected in the question 'Do you work in the healthcare sector?'

Yes, several times

Yes, once

No

Do you use face masks at work in situations where doing so is recommended?

This question only appears if the option 'Yes' is selected in the question 'Do you work in the healthcare sector?'

More than 75% of the time

Around 50% of the time

Less than 25% of the time

Never

What kind of personal protective equipment do you use at work?

This question only appears if the option 'Yes' is selected in the question 'Do you work in the healthcare sector?'

Multiple answers are possible.

Surgical face mask

P3 respirator

Gloves

Own glasses

Protective goggles

Visor

Hairnet/hat/cap

Protective gown

Footwear

Other

How often do you use personal protective equipment (except gloves) at work?

This question only appears if the option 'Yes' is selected in the question 'Do you work in the healthcare sector?'

Often Daily

Once daily

Weekly

Monthly

Never

Do you know if you have been exposed to coronavirus in a work context?

This question only appears if the option 'Yes' is selected in the question 'Do you work in the healthcare sector?'

(for example if a coronavirus patient has coughed on you)

Yes, with the recommended protection

Yes, without the recommended protection

No

Don’t know

What health institution do you work at?

This question only appears if the option 'Yes' is selected in the question 'Do you work in the healthcare sector?'

Name and ward/department if applicable.

Approximately how many patients with confirmed coronavirus have been treated in your ward/department at work?

This question only appears if the option 'Yes' is selected in the question 'Do you work in the healthcare sector?'

None 1–5

6–10

11–20

21–50

More than 50

Have you personally given treatment to coronavirus patients in your work?

This question only appears if the option 'Yes' is selected in the question 'Do you work in the healthcare sector?'

Yes

No

Don’t know

I feel confident that my workplace will do their utmost to make sure I do not get infected at work.

This question only appears if the option 'Yes' is selected in the question 'Do you work in the healthcare sector?'

Rate the extent to which you agree on a scale of 0 (completely disagree) to 7 (completely agree)

0 completely disagree

1

2

3

4

5

6

7 completely agree Don’t know

Page break

Page 3

POSSIBLE CORONAVIRUS RISK FACTORS

Check off any illnesses or conditions you have.

You can tick one option per condition.

Chronic heart disease including congenital heart disease (not high blood pressure)

Yes

No

Don’t know

High blood pressure

Yes

No

Don’t know

Chronic lung disease (other than asthma)

Yes

No

Don’t know

Asthma

Yes

No

Don’t know

Diabetes

Yes

No

Don’t know

Immunosuppressive treatment

Yes

No

Don’t know

Cancer (being treated)

Yes

No

Don’t know

Other illnesses

Yes

No

Don’t know

Write in any other illnesses here:

What is your height in centimeters?

What is your weight in kilograms?

Are you a smoker?

Yes

No, I have never smoked

Yes, I was a smoker before

Yes, I vape

Don’t know

I would say my physical condition is:

Poor

Average

Good

How many times per month are you in contact with the healthcare sector as a patient?

For example, to visit your doctor, undergo a medical examination or to receive treatment.

0, I rarely have contact with the healthcare sector

1–2

3–4

More than 4

Are you pregnant?

Yes

No

Don’t know

Page break

Page 4

YOUR HABITS BEFORE NORWAY ENTERED LOCKDOWN ON MARCH, 13

The questions below refer to the period two weeks before Noway entered lockdown on March, 13.

This question only appears if the option 'Yes, at least one test came back positive and I have/had coronavirus' or 'Yes, but the result was negative' is selected in

the question 'I have been tested for coronavirus'.

If your first coronavirus test was taken before March, 13 then base your answer on the two weeks BEFORE you were tested/sick!

Select the number of times in this period you did the following things:

SHOPPING HABITS

Went to the grocery store.

Number of times in the period.

0

1–3

4–10

11 or more

Went to the grocery store and stood in line at the check-out.

Number of times in the period.

0

1–3

4–10

11 or more

Went to another type of store or shopping center?

Number of times in the period.

0

1–3

4–10

11 or more

Came within closer than one meter of others at a store or shopping center?

Number of shopping trips in the period.

0

1–3

4–10

11 or more

TRAVEL

The following questions are about travel in order to learn more about how coronavirus can be transmitted when traveling. The questions below refer to the period two weeks before Noway entered lockdown on March, 13.

This question only appears if the option 'Yes, at least one test came back positive and I have/had coronavirus' or 'Yes, but the result was negative' is selected in

the question 'I have been tested for coronavirus'.

If your first coronavirus test was taken before March, 13 then base your answer on the two weeks BEFORE you were tested/sick!

Select the number of times in this period you did the following things:

Used public transport.

For example the subway, bus, train, streetcar, domestic ferry (not taxis).

Number of times in the period.

0

1–3

4–10

11 or more

Used public transport during rush hour.

Number of times in the period.

0

1–3

4–10

11 or more

Used public transport where you or others had to stand because all of the seats were taken.

Number of times in the period.

0

1–3

4–10

11 or more

Traveled abroad by plane.

Number of times in the period.

0

1–3

4–10

11 or more

What country/countries did you visit?

This question only appears if the option '1–3', '4–10' or '11 or more' is selected in the question 'Traveled abroad by plane'.

Traveled domestically by plane.

Number of times in the period.

0

1–3

4–10

11 or more

Traveled abroad by ferry or cruise.

Number of times in the period.

0

1–3

4–10

11 or more

Page break

Page 5

YOUR CONTACT WITH OTHERS

The questions below refer to the period two weeks before Norway entered lockdown on March, 13.

This question only appears if the option 'Yes, at least one test came back positive and I have/had coronavirus' or 'Yes, but the result was negative' is selected in

the question 'I have been tested for coronavirus'.

If your first coronavirus test was taken before March, 13 then base your answer on the two weeks BEFORE you were tested/sick!

Select the number of times in this period you did the following things:

How many times were you in a crowd or a place with 4–9 other people from outside your household?

For example a work meeting, gathering, restaurant, pub/bar, concert, gym, sporting event or similar – not including gatherings outside.

Number of times in the period.

0

1–3

4–9

10 or more

How many times were you in a crowd or a place with 10–50 other people?

For example a work meeting, gathering, restaurant, pub/bar, concert, gym, sporting event or similar – not including gatherings outside.

Number of times in the period.

0

1–3

4–9

10 or more

How many times were you in a crowd or a place with more than 50 other people?

For example a work meeting, gathering, restaurant, pub/bar, concert, gym, sporting event or similar – not including gatherings outside.

Number of times in the period.

0

1–3

4–9

10 or more

Been in contact with children (closer than one meter) who do not live in your household?

For example when babysitting or in a work context.

Number of times in the period.

0

1–3

4–9

10 or more

Page break

YOUR HABITS AFTER NORWAY ENTERED LOCKDOWN ON MARCH, 13

Page 6

This question only appears if the option 'Yes, at least one test came back positive and I have/had coronavirus' or 'Yes, but the result was negative' is selected in

the question 'I have been tested for coronavirus'.

The questions below apply to the last two weeks

This question only appears if the option 'Yes, at least one test came back positive and I have/had coronavirus' or 'Yes, but the result was negative' is selected in

the question 'I have been tested for coronavirus'.

If your first coronavirus test was taken after March, 13 then base your answer on the period between March, 13 and when you were tested/got sick.

Select the number of times in this period you did the following things:

SHOPPING HABITS

Went to the grocery store.

Number of times in the period.

0

1–3

4–10

11 or more

Went to the grocery store and stood in line at the check-out.

Number of times in the period.

0

1–3

4–10

11 or more

Went to another type of store or shopping center?

Number of times in the period.

0

1–3

4–10

11 or more

Came within closer than one meter of others at a store or shopping center?

Number of shopping trips in the period.

0

1–3

4–10

11 or more

TRAVEL

The questions below apply to the last two weeks

This question only appears if the option 'Yes, at least one test came back positive and I have/had coronavirus' or 'Yes, but the result was negative' is selected in

the question 'I have been tested for coronavirus'.

If your first coronavirus test was taken after March, 13 then base your answer on the period between March, 13 and when you were tested/got sick.

Select the number of times in this period you did the following things:

Used public transport.

For example the subway, bus, train, streetcar, domestic ferry (not taxis)

Number of times in the period.

0

1–3

4–10

11 or more

Used public transport during rush hour.

Number of times in the period.

0

1–3

4–10

11 or more

Used public transport where you or others had to stand because all of the seats were taken.

Number of times in the period.

0

1–3

4–10

11 or more

Traveled abroad by plane.

Number of times in the period.

0

1–3

4–10

11 or more

What country/countries did you visit?

This question only appears if the option '4–10', '1–3' or '11 or more' are selected in the question 'Traveled abroad by plane'.

Traveled domestically by plane.

Number of times in the period.

0

1–3

4–10

11 or more

Traveled abroad by ferry or cruise.

Number of times in the period.

0

1–3

4–10

11 or more

Page break

Page 7

YOUR CONTACT WITH OTHERS AFTER MARCH, 13

The questions below apply to the last two weeks

This question only appears if the option 'Yes, at least one test came back positive and I have/had coronavirus' or 'Yes, but the result was negative' is selected in

the question 'I have been tested for coronavirus'.

If your first coronavirus test was taken after March, 13 then base your answer on the period between March, 13 and when you were tested/got sick.

Select the number of times in this period you did the following things:

How many times were you in a crowd or a place with 4–9 other people from outside your household?

For example a work meeting, gathering, restaurant, pub/bar, concert, gym, sporting event or similar – not including gatherings outside.

Number of times in the period.

0

1–3

4–9

10 or more

How many times were you in a crowd or a place with 10–50 other people?

For example a work meeting, gathering, restaurant, pub/bar, concert, gym, sporting event or similar – not including gatherings outside.

Number of times in the period.

0

1–3

4–9

10 or more

How many times were you in a crowd or a place with more than 50 other people?

For example a work meeting, gathering, restaurant, pub/bar, concert, gym, sporting event or similar – not including gatherings outside.

Number of times in the period.

0

1–3

4–9

10 or more

Been in contact with children (closer than one meter) who do not live in your household?

For example when babysitting or in a work context.

Number of times in the period.

0

1–3

4–9

10 or more

Page break

YOUR HOUSEHOLD

Page 8

Coronavirus can be transmitted among people who live together. In China, this was a very significant route of transmission. The questions below aim to establish how significant this route of transmission is in Norway.

How many people live in your household? Number of people including yourself (anyone you share a bathroom and/or kitchen with)

1

2

3

4

5

6

More than 6

Has anyone in your household other than yourself been in quarantine or home isolation due to coronavirus in the last three weeks?

This question only appears if option '2', '3', '4', '5', '6' or

'More than 6' is selected in the question 'How many people live in your household?'

The question refers to quarantine as per national guidelines or a period of quarantine ordered by a doctor/the authorities. For example after traveling abroad and displaying symptoms of coronavirus or after having been potentially exposed to the infection.

Yes No

Don’t know

When did your fellow household member(s) begin their qurantine/home isolation?

This question only appears if the option 'Yes' is selected in the question 'Has anyone in your household other than yourself been in quarantine or home isolation

due to coronavirus in the last three weeks?'

Give the start date of the first quarantine period in the case several people have been in quarantine.

When did your fellow household member(s) end their quarantine/home isolation?

This question only appears if the option 'Yes' is selected in the question 'Has anyone in your household other than yourself been in quarantine or home isolation

due to coronavirus in the last three weeks?'

Leave empty if they are still in quarantine/home isolation.

Check off every symptom that others in your household have had over the last three weeks:

This question only appears if option '2', '3', '4', '5', '6' or

'More than 6' is selected in the question 'How many people live in your household?'

Multiple answers are possible. If several people in your household have had symptoms, check off all symptoms that anyone in your household has had.

Fever

High fever (over 39)

Shortness of breath

Cough

Tiredness/exhaustion

Muscle pains

Sore throat

Impaired sense of smell/taste

Blocked or runny nose

Headache

Stomach pains/nausea/diarrhea

Other symptoms

No symptoms

Elaborate on what other symptoms members of your household have had:

This question only appears if the option 'Other symptoms' is selected in the question 'Check off every symptom that others in your household have had

over the last three weeks:'

When did your household member(s) begin experiencing symptoms?

This question only appears if at least one of the options 'High fever (over 39)', 'Other symptoms', 'Shortness of breath', 'Muscle pains', 'Cough',

'Fever', 'Sore throat', 'Blocked or runny nose', 'Stomach pains/nausea/diarrhea',

'Tiredness/exhaustion', 'Impaired sense of smell/taste' or 'Headache' is selected in the question 'Check off every symptom that others in your household have had over the last three weeks:'

Give the date for when symptoms began in the first person to experience symptoms.

When did your household member(s) stop experiencing symptoms?

This question only appears if at least one of the options 'High fever (over 39)', 'Other symptoms', 'Shortness of breath', 'Muscle pains', 'Cough',

'Fever', 'Sore throat', 'Blocked or runny nose', 'Stomach pains/nausea/diarrhea',

'Tiredness/exhaustion', 'Impaired sense of smell/taste' or 'Headache' is selected in the question 'Check off every symptom that others in your household have had over the last three weeks:'

Leave empty if anybody in your household is still experiencing symptoms.

How many children (under the age of 18) live in your household?

0

1

2

3

4

5

More than 5 children

Do any of the children have shared residence (i.e. alternate between living with their mom/dad)?

This question only appears if the option '1', '2', '3', '4', '5' or 'More than 5 children' is selected in the question 'How many children (under the age of 18) live

in your household?'

Yes

No

Enter the child’s age in years

This question only appears if the option '1', '2', '3', '4', '5' or 'More than 5 children' is selected in the question 'How many children (under the age of 18) live

in your household?'

Enter the child’s age in years

This question only appears if the option '2', '3', '4', '5', '5' or 'More than 5 children' is selected in the question 'How many children (under the age of 18) live in your

household?'

Enter the child’s age in years

This question only appears if option '4', '5', or 'More than 5 children' is selected in the question 'How many children (under the age of 18) live in your household?'

Enter the child’s age in years

This question only appears if option '3', '4', '5' or 'More than 5 children' is selected in the question 'How many children (under the age of 18) live in your household?'

Enter the child’s age in years

This question only appears if option '5' or 'More than 5 children' is selected in the question 'How many children (under the age of 18) live in your household?'

Enter the child’s age in years

This question only appears if the option 'More than 5 children' is selected in the question 'How many children (under the age of 18) live in your household?'

Enter the ages of the other children

This question only appears if the option 'More than 5 children' is selected in the question 'How many children (under the age of 18) live in your household?'

This question only appears if the option '1', '2', '3', '4', '5' or 'More than 5 children' is selected in the question 'How many children (under the age of 18) live

in your household?'

Children rarely become seriously ill from coronavirus but it is unknown whether they help spread the infection to others. This is something that we are attempting to ascertain through the questions below.

Have your school-aged children been kept home from school since Norway entered lockdown on March, 13?

This question only appears if the option '1', '2', '3', '4', '5' or 'More than 5 children' is selected in the question 'How many children (under the age of 18) live

in your household?'

Yes

Partly.

The children have been to school a bit

No

Not applicable

Have your kindergarten-aged children been kept home from kindergarten since Norway entered lockdown on March, 13?

This question only appears if the option '1', '2', '3', '4', '5' or 'More than 5 children' is selected in the question 'How many children (under the age of 18) live

in your household?'

Yes

Partly.

The children have been to kindergarten a bit

No

Not applicable

Have your children been in close contact or played with other children (outside your household) after March, 13?

This question only appears if the option '1', '2', '3', '4', '5' or 'More than 5 children' is selected in the question 'How many children (under the age of 18) live

in your household?'

Close contact is defined here as

more than one minute and either indoors or within a distance of one meter outdoors. For example, playing together in the playpark.

Yes, the children have played with more than 10 other children.

Yes, the children have played with 3–10 other children

Yes, the children have played with 1–2 other children.

No, the children have not played with anyone outside of the household.

Have you got pets at home?

Yes, dog

Yes, cat

Yes, other

No

What type of property do you live in?

Apartment

Terraced house

Semi-detached house

Detached house

Other

Do you live in a town/city?

Yes

No

Where does your partner work (if applicable)?

In the healthcare sector with direct patient contact

In the healthcare sector without patient contact

Store/pharmacy/shopping center

Transport sector

Teacher at a school/after-school center or employee in a kindergarten

Other

Not applicable

Has your partner been working from home after March, 13?

Yes

No

Partly

Not applicable

Page break

YOUR PLACE OF WORK / STUDY

Page 9

Before March, 13: How many people did you have close contact with on an average day at work/school?

Close contact is, for example, being in the same room for more than one minute, sharing an elevator or being within one meter from each other in a large room.

0

1–2

3–10

11–30

More than 30

Not applicable

After March, 13: How many people did you have close contact with on an average day at work/school?

Close contact is, for example, being in the same room for more than one minute, sharing an elevator or being within one meter from each other in a large room.

0

1–2

3–10

11–30

More than 30

Not applicable

In what sort of an environment do you mostly work?

Multiple answers are possible.

In an office (alone)

Home office

In an open-plan/shared office

Restaurant/bar/reception desk or similar

As a driver

Directly with people (patients/customers/students or similar)

In a store

As a cleaner

Retired

Warehouse

Outdoors

Mostly alone

As a tradesman

I am not in work

What is your working situation now?

This question only appears if at least one of the options 'As a driver', 'Mostly alone', 'Directly with people (patients/customers/students or similar)',

'As a tradesman', 'In a store', 'As a cleaner', 'Retired', 'Warehouse',

'Outdoors', 'In an office (alone)' or 'In an open-plan/shared office' is selected in the question 'In what sort of an environment do you mostly work?'

Full-time

Part-time

Furloughed Not in work

Have you been working at home since the schools closed on March, 13?

Yes, full time.

Partly

I have been going to work as normal.

Not applicable

Does your place of work/study practice the quarantine rules strictly or are exemptions made to the rules?

The quarantine rules are followed closely

The quarantine rules are followed to an extent

The quarantine rules are not followed

Not applicable

State what your job is (if applicable):

For example taxi driver, waiter, store assistant, food delivery driver, school or kindergarten employee, etc.

Page break

Page 10

OTHER QUESTIONS

How many times do you wash your hands per day?

0

1–2

3–6

7–12

More than 12 times

How many times per day do you disinfect your hands (use antibac)?

0

1–2

3–6

7–12

More than 12 times

How many times have you suffered from a mild infection over the last 6 months?

For example the common cold, influenza or a urinary tract infection without hospitalization.

0

1–2

3–4

5–6

More than 6 times

What health food products or dietary supplements do you take more often than once per week? Include any nasal sprays (such as Carragelose products).

How many different medicines do you take regularly?

0

1

2

3

4

5

6

7

8

9

More than 9

Write in the names of those you remember:

Have you been using a face mask or respirator outside of work?

Every time I leave the house

Sometimes when I leave the house

Never

What type of face mask have you used?

This question only appears if the option 'Every time I leave the house' or 'Sometimes when I leave the house' is selected in the question 'Have you been

using a face mask or respirator outside of work?'

Multiple answers are possible.

Fabric face mask

Sports or pollen face mask

Surgical face mask

Respirator, not sure what type

P1 P2 P3 N95 N99

Paper respirator

Half rubber respirator

Full face respirator

Scarf around the mouth purchased at a hardware store

Purchased at a pharmacy

Purchased online

Have you been using gloves to protect against infection outside of working hours?

Every time I leave the house

Sometimes when I leave the house Never

Do you normally wear glasses?

Yes, often On and off Never

How many times per day have you left your home over the past week?

Not including your own yard/garage or when checking the mailbox/taking out the trash.

0

1–2

3–4

5 or more

Enter your age

Sex

Male

Female

Other

Is it okay if we get back to you to ask about taking a blood or respiratory sample?

A respiratory sample can be used to find the virus and involves using a thin swab in the mouth and/or nose.

Yes

No

Would you like to receive information about the results of this survey via email?

Yes

No

Your comments on the form or the coronavirus situation:

See new changes in Nettskjema (v924_0
